# News and Notes

**Published:** 1904-04

**Authors:** 


### *NEWS AND NOTES.*

IT will be of interest to all physicians in the State to learn that a professional nurses' register has been established at Palmer's Viaduct Pharmacy, 11 Peachtree street, Atlanta. Only graduates and competent names are on this register, and one can be obtained by either writing, telegraphing or telephoning them. Phone 64.

### THE PRUNE.

What is that thing, dark-blue and thick,
Just like a blood-distended tick,
That makes the boarders' heart turn sick ?
The prune.

It has its place at every meal,
What odors, nauseating, steal
That makes the stomach turn and reel—
The prune.

At lunch, at dinner, as sure as fate,
I'll find it sitting by my plate.
It makes my bile coagulate—
The prune.

Whate'er its culinary guise,
Stewed in pots or baked in pies,
It's only fit to draw the flies—
The prune.

It's used, they say, in certain bum
Wet goods, whiskey and rum,
And also in prune juice sputum—
The prune.

Some day, when I've got lots of dough,
I'll try and buy the whole prune show,
And then into the sewer you'll go—
You prune!

DR. W. A. CROW has been appointed Gynecologist to the Grady Hospital to succeed the late Dr. V. O. Hardon.

---

THE second Latin-American Congress of Medicine and Hygiene will be held in Beunos Ayres, April 3 to 10, 1904.

---

DR. H. P. COOPER has been elected to the chair of obstetrics and gynecology in the Atlanta College of Physicians and Surgeons made vacant by the death of Dr. V. O. Hardon.

---

IT is stated that Dr. Nicholas Senn, of Chicago, Ill., will shortly go to Japan to assume charge of the medical and surgical corps of the Mikado's army during the current war with Russia.

---

ALTHOUGH Mr. Curie has been honored with a professorship in the faculty of science in Paris, money and space were lacking at the Sorbonne to furnish him with an adequate laboratory. The Pasteur Institute has come to the rescue with the required space and apparatus.

---

A VALUABLE local anesthetic for inflamed tissues can be had by adding a few drops of 1:1000 solution of adrenalin to a half- to one-per-cent. solution of cocaine. In injecting this the first result is an anemia of the skin, due to its action on the vasoconstrictors, followed by a deep anesthesia. Incisions can be made into inflamed tissues with the loss of very little blood, and the method is well adapted for furuncles, anthrax, etc.—*Medical Record.*

---

AT the recent Tuberculosis Exposition at Baltimore, the following rules for consumptives attracted considerable attention: "Live in fresh air constantly; do not be afraid of cold or damp weather. Be outside all the sunny hours of the day. Avoid overheated and ill-ventilated rooms, and keep the windows of your bedroom open all night. If you avoid a draught of air you need not fear a cold.

Do not overclothe yourself; wear woolen garments next the skin, but do not wear more clothes than healthy people wear. A cold sponge bath every morning will make you less liable to take cold. Drink much milk and eat as much as possible, even if you do not care to."

---

THE first International Congress of School Hygiene is to be held at Nuremberg, April 4 to 9. Addresses are to be made on "What has Ophthalmology Done for the School-children, and What Has it Yet to Do?" by H. Cohn, of Breslau; on "The Present Status of School Hygiene in Norway," by A. Johannesen, of Christiania; on the "Hygiene and Personal Illness of the Teachers from the Standpoint of their Relations to their Pupils," by Le Gendre, of Paris; on "Organization of the Public Schools according to the Natural Capacity of the Children," by Sickinger, of Mannheim; on the "Task and Training of School Inspectors," by Liebermann, of Budapest; on "Prevention of Infectious Diseases in Schools," and on "Suicides Among School-children," by Eulenburg, of Berlin.

---

DR. SHELDON, of Madison, Wis., says the *Medical Record* is reported in the New York *Times* as a propounder of the theory that appendicitis is a contagious disease of microbic origin. This hypothesis, he believes, offers the only explanation of the epidemic of the disease which he says now prevails in the country. He would doubtless hold as confirmatory of this theory the story of three sisters in Augusta, Ga., who were all operated on for appendicitis in one day. The three girls were students at an academy in Washington, Ga. One was attacked on Saturday, another on Sunday, and the third on Monday, and the three operations were performed on Tuesday. They were all brought to Augusta for the operations. The father of the girls was himself operated on for appendicitis a few months ago.

---

AN exchange states that Prof. Robert Koch, the German scientist, has written from South Africa a letter to a member of the Committee on the Prevention of Tuberculosis of the New York Charity Organization Society, expressing great satisfaction that at last some one has arisen to interfere with the swindlers who have been trading upon his name and reputation. He says: "For my part, whenever opportunity offered itself, I have never neglected to declare that I had nothing whatever to do with Koch Lung Cure, and that these concerns used my name for fraudulent purposes. I would long since have brought suit against these swindlers, but friends familiar with the conditions in America advised against it, saying that according to the law in the United States these imposters could not be prosecuted. I, too, begin to believe that the only rational way to deal with them is the one taken by your committee on the prevention of tuberculosis. The public should be informed through the press of the outrageous manner in which these unscrupulous people cheat and fleece the unfortunate tuberculous."

---

THE fifty-fifth annual session of the Medical Association of Georgia will be held at Macon, Ga., April 20, 21 and 22, 1904. Preliminary program:

Some Dangers to the Public Health of Georgia Needing Control by Legislation. R. M. Harbin, M.D., Rome.

The Treatment of Cancer. M. B. Hutchins, M.D., Atlanta.

The Moral and Religious Side of the Medical Profession. E. Morgan, M.D., Vidalia.

A Plea for a More Thorough Examination and Rational Treatment of Nose and Throat Disease. Dunbar Roy, M.D., Atlanta.

Antiseptic and Eliminative Treatment of Typhoid Fever. T. V. Hubbard, M.D., Atlanta.

Two Cases of Ectopic Pregnancy Going to Term—Operation, Recovery. E. R. Corson and J. G. Van Marten, M.D., Savannah.

Some Frequently Misunderstood Symptoms of General Interest in the Region of the Head. A. W. Stirling, M.D., Atlanta.

Consumption and The State. M. M. Saliba, M.D., Savannah.

The Clinical Consideration of Tumors. W. F. Westmoreland, M.D., Atlanta.

Hysterectomy for Fibroid Tumors of the Uterus. Geo. H. Noble, M.D., Atlanta.

Anesthesia and Anesthetists. Ralph M. Thomson, M.D., Savannah.

The Prophylaxis of Venereal Diseases. W. L. Champion, M.D., Atlanta.

When to Circumcise the Infant if Not on the Eighth Day. S. A. Visanska, M.D., Atlanta.

The Best Operation for Vesical Calculus, with Report of Cases. M. F. Carson, M.D., Griffin.

It's Only a Running Ear. J. M. Crawford, M.D., Atlanta.

How We Should Treat Rheumatism. J. W. Palmer, M.D., Ailey.

Renal Decapsulation—Indications, Limitations and Technique. R. R. Kime, M.D., Atlanta.

A Few Surgical Cases. O. T. Kenyon, M.D., Dawson.

The Attic of the Nose, with Some Cases of Aural and Brain Complication. A. G. Hobbs, M.D., Atlanta.

Tuberculosis. J. E. Sommerfield, M.D., Atlanta.

The Specific Treatment of Morphinism. C. C. Stockard, M.D., Atlanta.

Ectopic Gestation, with Report of Complete Operation, with Recovery of Patient. E. C. Davis, M.D., Atlanta.

Uncinariasis in Georgia. Claude A. Smith, M.D., Atlanta.

Clinic of Deaf Mutes, whose Hearing and Speech Have Been Established and the Instrument and Technic Applicable to Various Diseases and Deaf Conditions. M. M. Stapler, M.D., Macon.

